# Phytochemical-based quality and marker identification of sweet tea (*Lithocarpus litseifolius* [hance] Chun) from various Jiangxi regions

**DOI:** 10.1016/j.fochx.2026.104049

**Published:** 2026-05-30

**Authors:** Yuling Wang, Bing Cao, Jianfeng Cheng, Mengxing Wang, Zixuan Qiu, Wuping Yan

**Affiliations:** a School of Breeding and Multiplication (Sanya Institute of Breeding and Multiplication), Hainan University, Sanya 572025, China; b School of Agricultural Sciences, Jiangxi Agricultural University, Nanchang 330045, China; c Hainan Academy of Agricultural Sciences, Haikou 570203, China

**Keywords:** Sweet tea, Dihydrochalcone, Flavonoid, Phenylpropanoid, Environmental factor, Geographic origin tracing

## Abstract

*Lithocarpus litseifolius* is a novel food ingredient with medicinal, tea, and natural sweetening properties. This study examines the quality traits and chemical composition of 10 *L. litseifolius* samples from different geographic origins. Key metabolites are more concentrated in high-quality core regions, with Hengfeng having the highest levels and Anfu the lowest. Of the total 621 metabolites, 52 were significantly differentially accumulated and enriched in “metabolic pathways” and “secondary metabolite biosynthesis.” Unsupervised hierarchical clustering yielded two distinct clusters, aligning with established quality grades and geographical proximity. Mantel correlation and distance-based redundancy analysis identified soil available copper, phosphorus, potassium, and iron, as well as mean annual temperature, atmospheric pressure, and humidity, as key drivers of regional differences. Random forest ranking and PCA-based dimensionality reduction identified eight metabolites as markers of geographical origin. This study demonstrates a soil and climate-driven reconfiguration of secondary metabolism in *L. litseifolius*, offering guidance for region-based cultivation optimization.

## Highlights

• *Lithocarpus litseifolius* chemical quality exhibits significant geographical variation.
• Non-targeted metabolomic analysis reveals distinct geography-based chemical profiles.
• PTW origin is potential high-quality germplasm resource with superior overall quality.
• Soil available copper, iron and temperature are key environmental factors for metabolite variation.
• Eight metabolites are identified as potential biomarkers for geographical origin tracing.

## 1 Introduction

*Lithocarpus litseifolius* [Hance] Chun, of the genus *Lithocarpus* within the family Fagaceae, is a valuable plant resource for medicinal, nutritional, and tea-processing applications (Sun et al., 2025). Its young leaves and branches have a natural sweetness, leading to its widespread designation as “sweet tea.” This species is primarily distributed in mountainous regions south of the Yangtze River in China (Guo et al., 2021), with the highest concentration in the Wuyi Mountains at the junction of Jiangxi and Fujian provinces. Local communities commonly harvest young buds and leaves and process them into tea products using techniques analogous to those employed for green or black tea production, resulting in an estimated annual output exceeding 2000 metric tons (Wang et al., 2022). In 2017, *L. litseifolius* was officially approved by the National Health Commission of China (formerly the National Health and Family Planning Commission) as a novel food ingredient and is widely utilized as a natural sweetener in the food industry (Liu et al., 2022).

Modern pharmacological studies have confirmed its favorable safety profile for human consumption and range of biological activities, including antihypertensive, hypolipidemic, hypoglycemic, antioxidant, anti-inflammatory, and anticancer effects (Shang et al., 2022; Wang et al., 2021), earning it the colloquial designation “tree Cordyceps.” Its therapeutic potential is largely attributed to its rich array of secondary metabolites, particularly flavonoids, triterpenoids, and phenolic compounds (Cheng et al., 2018; Wang et al., 2021). Notably, dihydrochalcone (DHC) flavonoids, such as phlorizin, trilobatin, and phloretin, are the most characteristic bioactive constituents of *L. litseifolius*, exhibiting marked efficacy for the prevention and management of chronic conditions such as diabetes and hypertension (Guo et al., 2021; Shang et al., 2022). The accumulation levels of these metabolites directly determine the health benefits and overall quality of sweet tea. Owing to its exceptional nutritional and pharmacological properties, *L. litseifolius* has considerable promise for functional food and pharmaceutical applications, with a steadily increasing market demand.

The pharmacological quality of medicinal plants is primarily determined by the types and concentrations of their secondary metabolites, which often vary significantly across geographic origins (Li et al., 2020; Yu et al., 2024). These variations arise from complex interactions among environmental factors, such as temperature, light intensity, precipitation, soil composition, and plant physiological responses during growth, which jointly regulate key biosynthetic pathways, thereby influencing secondary metabolite accumulation (Wang et al., 2024; Yang et al., 2024). The biological effects of *L. litseifolius*, a plant valued for both its medicinal and edible uses, are largely due to the types and amounts of secondary metabolites it contains—especially flavonoids. Liu et al. (2025) demonstrated that the developmental stage and growth environment significantly influence metabolite accumulation in this species. Notably, samples from Jianghua County in Hunan Province have significantly higher phlorizin levels than those from other regions, whereas specimens from Hengfeng County in Jiangxi Province have elevated trilobatin and phloretin concentrations. Wang et al. (2021) conducted qualitative and quantitative analyses, comparing chemical constituents among different *L. litseifolius* varieties, and revealed substantial chemotypic differences that positively correlate with biological activities. Jiangxi Province, located in the subtropical humid monsoon zone of southern China, has an extensive mountainous and hilly terrain, with particularly concentrated resources in the region surrounding the northern foothills of the Wuyi Mountains, reflecting pronounced regional diversity.

Our research group previously evaluated the genetic diversity and quality phenotypes of *L. litseifolius* across the primary cultivation regions in Jiangxi Province (Luo, 2025; Wan, 2022). By integrating biochemical profiling and molecular marker analyses, a preliminary germplasm classification framework was developed, revealing limited intraspecific genetic diversity, extensive gene flow, and minimal population differentiation throughout the province. A comprehensive multi-trait evaluation identified Hengfeng (PTW), Yiyang (QG), Suichuan (FGS), Zixi (DJS), Xunwu (NFS), and Luxi (MYX) as core high-quality production areas, while Fenyi (NC), Jinggangshan (YLZ), Yuanzhou (SKC), and Anfu (HKC) were classified as general production areas. Nevertheless, it remains unclear whether geographically stable and chemically distinct metabolite profiles—particularly those mechanistically associated with key quality traits—are present and to what extent climatic and edaphic factors quantitatively influence their development. This knowledge gap hinders progress toward three translational objectives: (i) evidence-based selection of elite germplasm; (ii) establishment of a mechanism-driven authenticity assessment framework; and (iii) development of ecologically optimized, quality-assured cultivation strategies.

Given the complexity of its metabolic composition, a comprehensive analytical approach is required, integrating ultra-high-performance liquid chromatography–tandem mass spectrometry (UHPLC-MS/MS) and gas chromatography–mass spectrometry (GC–MS). Non-targeted metabolomic profiling can be achieved using UHPLC-MS/MS and GC–MS, whereas major dihydrochalone (DHC) constituents—including phlorizin, trilobatin, and phloretin—require ultra-high performance liquid chromatography coupled with triple quadrupole mass spectrometry (UHPLC-QqQ-MS). UHPLC-MS/MS is particularly suitable for detecting highly polar, thermally labile, and high-molecular-weight compounds, such as flavonoids, phenolic acids, saponins, and alkaloids, and is more effective for analyzing volatile and semi-volatile low-molecular-weight metabolites, including monoterpenes, sesquiterpenes, certain alkaloids, fatty acids, organic acids, and sugars (Hong et al., 2016; Liang et al., 2024). Integrating these two complementary techniques enables comprehensive coverage across a broad spectrum of metabolites, from polar to nonpolar and volatile to nonvolatile, minimizing information loss due to the inherent limitations of single-platform analyses, resulting in more complete and reliable data for the systematic characterization of plant chemical constituents and their functional properties (Nie et al., 2025; Shan et al., 2024). Furthermore, UHPLC-QqQ-MS, operated in multiple reaction monitoring (MRM) mode, enables highly sensitive and accurate quantification of key bioactive compounds, such as trilobatin and phlorizin (Liu et al., 2025).

Through this study, we sought to elucidate the patterns of variation in *L. litseifolius* quality traits and chemical compositions across geographical origins. Additionally, we investigated potential associations between environmental variables and quality, and provided a theoretical framework for germplasm screening, regional cultivation, and enhanced utilization. The findings support the development of geographical indications and promote the sustainable use of natural phytochemicals.

## 2 Materials and methods

### 2.1 Plant materials and experimental design

**Fig. 1:**
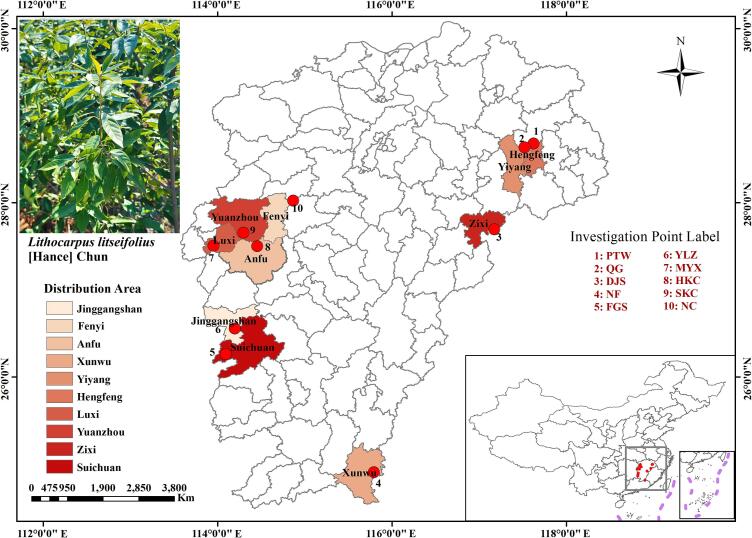
Geographic distribution of the 10 *Lithocarpus litseifolius* sampling locations in Jiangxi province, China.

To systematically assess geographical variation in *L. litseifolius* quality, field surveys were conducted across 10 production sites in Jiangxi Province during the spring of 2024; leaf and rhizosphere soil samples were collected from each site (Fig. 1). These 10 production sites encompass the main cultivation areas of *L. litseifolius* in Jiangxi Province. According to previous research, these locations can be divided into core high-quality production zones (Group 1: PTW, QG, NFS, DJS, MYX, and FGS) and general production zones (Group 2: NC, SKC, YLZ, and HKC). Sampling occurred exclusively during the spring leaf-expansion peak—considered the optimal harvest period for sweet tea quality processing. To maintain physiological consistency and minimize diurnal fluctuations in metabolite levels throughout the day, all samples were collected under cloudless conditions between 08:00 and 10:00 h. At each location, at least nine individual plants aged five years were selected, and leaves along with rhizosphere soil from every three plants were pooled to form one biological replicate; three replicates per site were prepared to ensure representative data and reliable results. After collection, leaf samples were immediately flash-frozen in liquid nitrogen and subsequently lyophilized until completely dry. The resulting freeze-dried powder was uniformly processed for metabolite extraction. Rhizosphere soil samples were air-dried under dark, clean, cool, and well-ventilated conditions to minimize alterations in physicochemical properties due to light exposure, temperature fluctuations, or humidity changes. Once fully dried, the samples were used for the subsequent analysis of soil physicochemical characteristics.

### 2.2 Sample preparation

For UHPLC-QqQ-MS/MS analysis, approximately 50 mg of sample was accurately weighed and extracted with 400 μL of a 75% methanol solution. The mixture was vortexed for 60 s, followed by homogenization using a tissue grinder at 55 Hz for 12 cycles. Extraction was performed in an ice bath for 30 min. Subsequently, the sample was centrifuged at 17,000 ×*g* for 15 min at 4 °C, and the supernatant was collected and stored on ice for subsequent use.

For UHPLC-MS/MS analysis, an appropriate amount of sample was precisely weighed into a 2 mL centrifuge tube and extracted with 600 μL of a methanol solution containing 4 ppm chlorophenylalanine as an internal standard. The mixture was vortexed for 30 s, and steel beads were added before homogenization in a tissue grinder at 55 Hz for 60 s. The extract was ultrasonicated at room temperature for 15 min. Afterward, the samples were centrifuged at 12,000 rpm for 10 min at 4 °C. The supernatant was filtered through a 0.22-μm organic phase syringe filter, and the filtrate was transferred to an autosampler vial for immediate UHPLC-MS/MS analysis.

For GC–MS analysis, an appropriate amount of sample was precisely weighed into a 2 mL centrifuge tube, and 0.5 mL of cold acetonitrile:isopropanol:water (1:1:1, v/v/v) extraction solvent was added with three stainless steel beads. The mixture was homogenized twice in a high-throughput tissue grinder at 60 Hz for 90 s. Subsequently, the samples were sonicated at room temperature for 5 min. The same cold solvent (0.5 mL) was then added, and sonication was continued for another 5 min. The extract was centrifuged at 12,000 rpm for 5 min at 4 °C, and 500 μL of the supernatant was transferred to a new 2 mL polypropylene microcentrifuge tube. The extract was concentrated to near-dryness under a vacuum using a centrifugal evaporator. Derivatization was carried out in two steps: first, 80 μL of 20 mg/mL methoxyamine hydrochloride in pyridine was added, followed by vortexing for 30 s and incubation at 60 °C for 60 min; second, 100 μL of *N*,*O*-bis(trimethylsilyl)trifluoroacetamide (BSTFA) containing 1% trimethylchlorosilane was added, followed by incubation at 70 °C for 90 min. After completing derivatization, the sample was centrifuged at 12,000 rpm for 5 min, and 90–100 μL of the upper phase was transferred to a glass insert within a GC autosampler vial for GC–MS analysis.

### 2.3 Quantitative analysis of DHC components

The major DHC compounds (phlorizin, trilobatin, and phloretin) in *L. litseifolius* leaves were quantitatively determined via UPLC-QqQ-MS/MS, following the method validation protocol described by Liu et al. (2025). The calibration curve consisted of seven concentration levels with good linearity (R^2^ > 0.995). The limits of detection and quantification were defined based on signal-to-noise ratios of 3:1 and 10:1, respectively. Both intra-day and inter-day precision, expressed as relative standard deviation (RSD), were below 5%, and spike recovery ranged from 80% to 120%. All analytical parameters met the quantitative criteria, verifying that this method is accurate and reliable. Briefly, the extract was filtered through a 0.22-μm organic-phase microporous membrane, and the filtrate was transferred to an HPLC autosampler vial for analysis. Phlorizin, phloretin, and trilobatin reference standards were individually dissolved in 75% methanol (v/v) to prepare single or mixed standard stock solutions, which were stored in the dark at −20 °C. Before analysis, the stock solutions were serially diluted with a 75% (v/v) methanol–water solution to generate a series of calibration working solutions across a defined concentration range. Each calibration point was analyzed under chromatographic conditions identical to those of the samples, and the peak areas of the target analytes were recorded. Using MultiQuant 3.0.3 software, calibration curves were constructed using the internal standard method, by plotting the response values against the corresponding concentrations of the standards, and these curves were subsequently used to determine target compound concentrations in the samples.

Chromatographic separation was achieved using a ZORBAX Rx-C8 column (1.8 μm, 4.6 mm × 50 mm) with a mobile phase composed of solvent A (0.1% formic acid in water) and solvent B (acetonitrile). The flow rate was 400 μL/min, the injection volume was 0.5 μL, the detection wavelength was 285 nm, and the column temperature was maintained at 40 °C. Mass spectrometric analysis was performed on an AB SCIEX Triple Quad™ 4500 mass spectrometer equipped with an electrospray ionization source operating in positive ion mode (electrospray ionization [ESI]+). Data acquisition was performed in MRM mode for both the precursor and product ions. Key ion source parameters were as follows: ion source gas temperature at 500 °C, curtain gas at 25 psi, collision gas at 10 psi, ion spray voltage at 4500 V, and nebulizer (source) temperature at 500 °C.

### 2.4 UHPLC-MS/MS analysis

The LC-MS/MS detection technique was conducted in accordance with the established analytical protocol outlined by Feng et al. (2025). Liquid chromatography was performed using a Vanquish ultra-high-performance liquid chromatography (UHPLC) system (Thermo Fisher Scientific, USA). Chromatographic separation was achieved with an ACQUITY UPLC® HSS T3 column (2.1 mm × 100 mm, 1.8 μm; Waters Corporation, Milford, MA, USA). The column temperature was maintained at 40 °C, the mobile phase flow rate was 0.3 mL/min, and the injection volume was 2 μL. For LC-ESI(+)-MS analysis, the mobile phase consisted of A2 (water containing 0.1% formic acid, v/v) and B2 (acetonitrile containing 0.1% formic acid, v/v). The gradient elution program was as follows: 0–1 min, 8% B2; 1–8 min, 8% → 98% B2; 8–10 min, 98% B2; 10–10.1 min, 98% → 8% B2; 10.1–12 min, 8% B2. For LC-ESI(−)-MS analysis, the mobile phase comprised solvent A3 (5 mM ammonium formate in water) and solvent B3 (acetonitrile), with a gradient profile identical to that used in positive ion mode: 0–1 min, 8% B3; 1–8 min, 8% → 98% B3; 8–10 min, 98% B3; 10–10.1 min, 98% → 8% B3; 10.1–12 min, 8% B3.

Metabolite detection via mass spectrometry was performed using an Orbitrap Exploris 120 mass spectrometer (Thermo Fisher Scientific, USA) equipped with an ESI source. Data were acquired in full-scan MS^1^ combined with data-dependent MS^2^ acquisition mode (Full MS ddMS^2^). Key instrument parameters were as follows: sheath gas pressure, 40 arb; auxiliary gas flow, 10 arb; spray voltage, +3.50 kV (positive mode) and − 2.50 kV (negative mode); capillary temperature, 325 °C; MS^1^scan range, *m*/*z* 100–1000; MS^1^resolution, 60,000 FWHM (at m/z 200); four data-dependent MS^2^scans triggered per duty cycle; MS/MS resolution, 15,000 FWHM (at m/z 200); normalized collision energy, 30%; dynamic exclusion duration, set to automatic mode.

### 2.5 GC–MS analysis

The GC–MS detection technique was conducted in accordance with the established analytical protocol outlined by Otify et al. (2023). Gas chromatography was performed using a Trace 1300 gas chromatograph (Thermo Fisher Scientific). Chromatographic separation was achieved using an Rxi-5Sil MS capillary column (30 m × 0.25 mm × 0.25 μm) with high-purity helium as the carrier gas at a constant flow rate of 1.0 mL/min. Split injection was employed with a split ratio of 20:1 and an injection volume of 1 μL; the injection port temperature was maintained at 280 °C. The ion source and transfer line interface temperatures were 300 °C and 280 °C, respectively. The oven temperature program was as follows: initial temperature of 50 °C held for 2 min; ramped to 180 °C at 5 °C/min; then further increased at 10 °C/min to 300 °C and held for 5 min. The total run time was 45 min. Metabolite detection was performed using an ISQ 7000 mass spectrometer (Thermo Fisher Scientific, USA) equipped with an electron impact ionization source. Data were acquired in full-scan mode (SCAN) at an electron energy of 70 eV.

### 2.6 Non-targeted metabolomic data processing and multivariate statistical analysis

For non-targeted metabolomic data analysis, raw mass spectrometry data files were initially converted to mzXML format using the MSConvert tool within the ProteoWizard software package (v3.0.8789). The XCMS package in R was used for peak detection, filtering, and alignment to generate a quantitative metabolite profile. The main parameters were configured as follows: bw = 2, ppm = 15, peakwidth = c (5, 30), mzwid = 0.015, mzdiff = 0.01, method = centWave. Subsequently, batch effects were eliminated by correcting the data using quality-control (QC) samples. Metabolites with RSDs greater than 30% in QC samples were excluded; only those remaining were used for further analysis. The resulting quantitative matrix was normalized using the total peak area method to minimize the systematic biases arising from instrument signal drift and variations in sample preparation. Metabolite identification was performed by integrating spectral matching with data from multiple public databases, including HMDB, MassBank, LipidMaps, MzCloud, KEGG, and an in-house standard compound database developed by Nomi Metabolomics. Multivariate statistical analyses were conducted using the R package ropls, including principal component analysis (PCA), partial least squares discriminant analysis (PLS-DA), and orthogonal partial least squares discriminant analysis (OPLS-DA). Model validity was assessed via permutation testing to prevent overfitting. For differentially accumulated metabolites (DAMs) selection, a combined criterion was applied based on statistically significant *P*-values (*P* < 0.05), the variable importance in projection (VIP > 1, derived from the OPLS-DA model), and fold-changes between groups, enabling a comprehensive evaluation of the contribution of each metabolite to group discrimination and biological interpretability. Functional pathway enrichment and topological analyses of the identified differential metabolites were performed using MetaboAnalyst (www.metaboanalyst.ca) to elucidate their potential biological roles.

### 2.7 Biochemical component analysis

The free amino acid (FAA) contents in the leaves of *L. litseifolius* were determined per the ninhydrin colorimetric method according to the protocol described by Zhang, Yang, et al. (2025). Tea polyphenol (TPs) contents were quantified using the ferric tartrate colorimetric method outlined by Peng et al. (2011). Tea pigments (TP) were quantitatively analyzed according to the method established by Lu et al. (2025). Theaflavin (TF) content was measured with the aluminum trichloride colorimetric assay described by Liang et al. (2022), with rutin employed as the calibration standard for quantification.

### 2.8 Determination of soil physicochemical properties

Rhizosphere soil samples were air-dried, ground, and passed through a 2-mm sieve. The organic matter (OM) content was determined using the potassium dichromate oxidation–external heating method (i.e., Walkley–Black method) (Wang et al., 2016). The soil pH was measured in a 1:2.5 (*w*/*v*) deionized water suspension after shaking for 1 h (Su et al., 2023), using a calibrated combined glass electrode. The concentrations of alkali-hydrolyzable nitrogen (HN), available phosphorus (AP), and available potassium (AK) were analyzed following previously established protocols (Zhang et al., 2019). To determine the available micronutrients, soil samples were extracted with a diethylenetriaminepentaacetic acid (DTPA) solution according to the procedure described by Graindorge et al. (2022). Specifically, 10 g of air-dried soil was shaken with 20 mL of DTPA extraction solution (0.005 mol/L DTPA, 0.01 mol/L CaCl₂, and 0.1 mol/L triethanolamine, adjusted to pH 7.3) for 2 h at room temperature. Following extraction, the mixture was filtered through a 0.45-μm membrane filter, and the filtrate was analyzed for available iron (AFe), exchangeable manganese (EMn), available copper (ACu), available zinc (AZn), and exchangeable magnesium (EMg) using inductively coupled plasma optical emission spectrometry (Optima 7300 DV, PerkinElmer).

### 2.9 Acquisition of climate factor data

Environmental factor data for each production area, including the mean annual temperature (MAT), mean annual precipitation (MAP), mean annual sunshine duration (MASD), mean annual wind speed (MAWS), mean annual atmospheric pressure (MAAP), mean annual humidity (MAH), and mean annual ground temperature (MAGT) at a depth of 20 cm were extracted using ArcGIS software based on global climate and weather data (https://www.worldclim.org) and meteorological data provided by the Jiangxi Provincial Meteorological Bureau.

### 2.10 Statistical analysis

The experimental data were analyzed using Origin 2021 (OriginLab Corp., Northampton, MA, USA) and SPSS 22.0 (IBM Corp., Armonk, N.Y., USA). Three biological replicates were collected at each sampling point, and three technical replicates were used for each sample. The results are presented as the mean ± standard deviation. One-way analysis of variance was performed to assess differences among groups, and Kendall's correlation coefficient was calculated to evaluate associations between variables. Statistical significance was set at *P* < 0.05, with differences considered statistically significant. The Pearson correlation coefficient was calculated using SPSS software (IBM Corp.,), and Mantel's test was used to investigate the relationships between key environmental factors and quality characteristics.

Distance-based redundancy analysis (dbRDA) was used to quantify the extent to which geographical distance independently accounts for variation in metabolite profiles. The Hellinger-transformed Bray-Curtis dissimilarity matrix was used as the response variable, while Euclidean distance derived from GPS coordinates acted as the only constrained predictor. Analyses were conducted in R (v.4.3.1) using the vegan package (v.2.6–4). Statistical significance was evaluated with 999 permutations (*P* < 0.05); the independent explanatory contribution of geography is reported as adjusted R^2^. Random Forest (RF) analysis was performed to determine the proportion of variance in *L. litseifolius* production area quality differentiation attributable to the identified DAMs. All analyses were performed with the R “randomForest” package, incorporating 1000 decision trees and using mean decrease in Gini impurity as the metric for variable importance ranking.

## 3 Results

### 3.1 DHC contents among regions

**Fig. 2:**
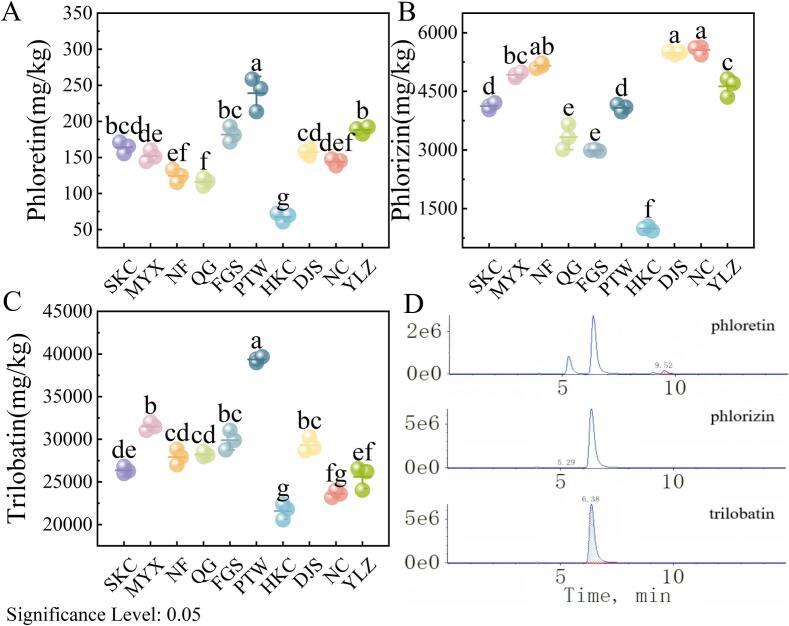
Contents of three key dihydrochalcones based on the 10 *Lithocarpus litseifolius* sampling locations in Jiangxi province, China. (A) Phloretin content. (B) Phlorizin content. (C) Trilobatin content. (D) The representative UPLC-QqQ-MS/MS typical chromatograms of test samples.

Significant differences in the levels of the three major DHCs were observed among the *L. litseifolius* samples of different geographical origins (*P* < 0.05; Fig. 2). That of the PTW origin had significantly higher concentrations of phloretin and trilobatin, reaching 239.17 ± 23.18 and 39,349.45 ± 338.67 mg/kg, respectively, compared to those of other origins. NC, DJS, and NF samples showed relatively high levels of phlorizin, all exceeding 5000 mg/kg. In contrast, HKC-origin samples had the lowest levels of phloretin (67.68 ± 6.11 mg/kg), trilobatin (21,583.71 ± 932.60 mg/kg), and phlorizin (990.05 ± 61.17 mg/kg) among samples of all origins, indicating distinct regional metabolic variation. Thus, samples collected from the six core high-quality production areas exhibited notably higher mean concentrations of the three DHCs than those from general production regions.

The Mantel test identified significant relationships between environmental variables and the accumulation of these DHCs (Fig. S1). In particular, phloretin and trilobatin levels were significantly positively correlated with soil ACu and MAWS (*r* > 0.21, *P* < 0.05). Additionally, trilobatin content exhibited significant positive correlations with soil AK, MAH, and MAT (*r* > 0.16, *P* < 0.05). These findings suggest that environmental factors, including temperature, light intensity, and soil composition, play crucial roles in regulating the accumulation of key bioactive constituents, such as phlorizin and trilobatin, which contribute to the characteristic flavor and health-promoting properties of *L. litseifolius*.

### 3.2 Biochemical quality components by origin

**Fig. 3:**
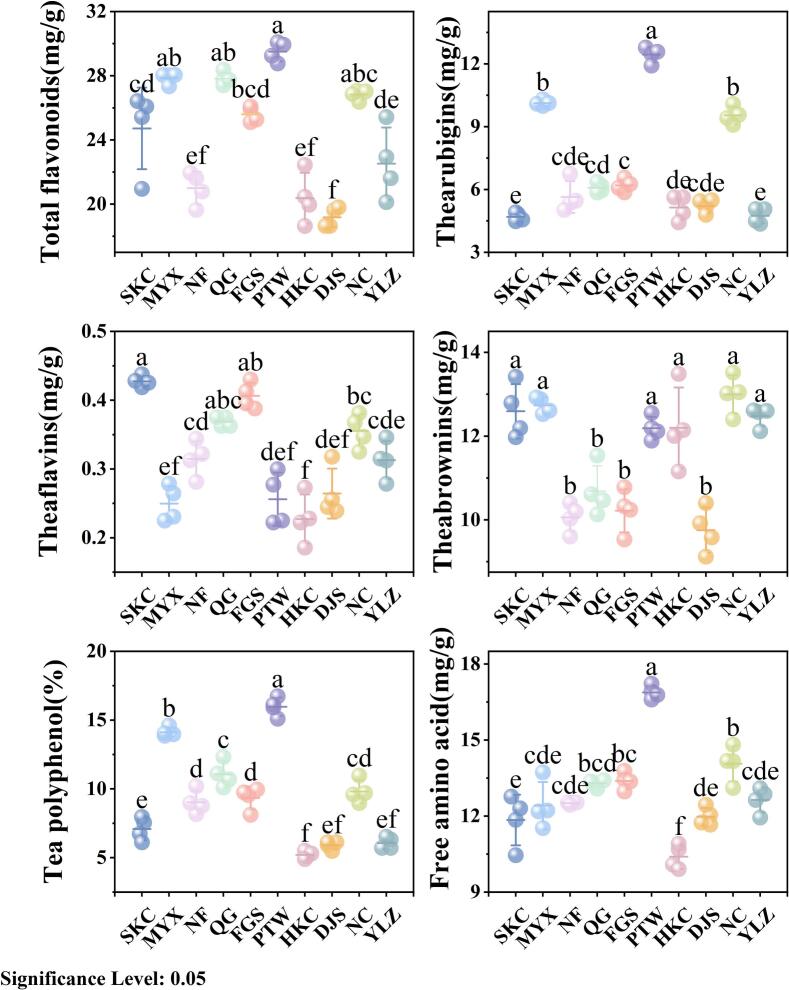
Biochemical quality differences among the 10 *Lithocarpus litseifolius* sampling locations in Jiangxi province, China.

Significant variations were observed in the biochemical components of tea samples with different origins (Fig. 3). PTW-origin samples had the highest total flavonoid (29.51 ± 0.61 mg/g), TR (12.43 ± 0.37 mg/g), total polyphenol (15.96 ± 0.67%), and FAA (16.88 ± 0.26 mg/g) levels, indicating superior overall tea quality; those of MYX and QG origins followed in performance. Samples of the SKC and FGS origins were distinguished by notably high theaflavin contents, whereas elevated theabrownin (TB) levels were primarily found in the NC, MYX, SKC, and YLZ samples. In comparison, the HKC sample exhibited the poorest comprehensive quality, with the lowest values for total polyphenols (5.20 ± 0.26%), free amino acids (10.40 ± 0.48 mg/g), and theaflavins (0.23 ± 0.04 mg/g) among all sampled regions.

### 3.3 Non-targeted metabolomic LC-MS/MS analysis

#### 3.3.1 Metabolic profiling of *L. litseifolius* from different origins

**Fig. 4:**
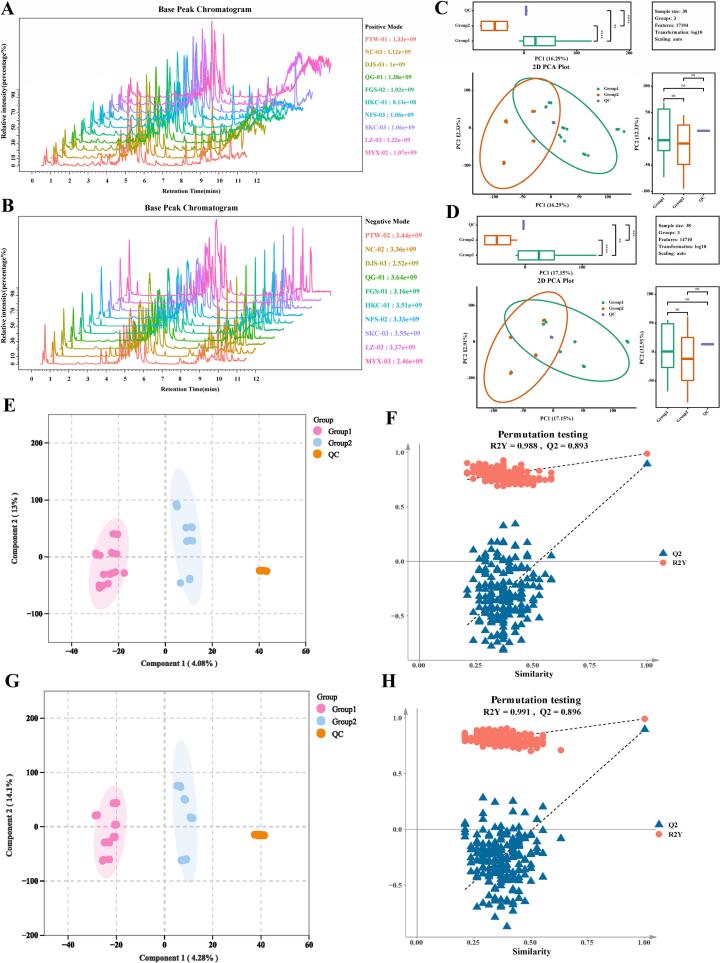
Multivariate statistical analysis of *Lithocarpus litseifolius* metabolites following LC-MS/MS detection across 10 sampling locations. (A) Base peak chromatogram (BPC) in positive ion mode. (B) BPC in negative ion mode. (C) Principal component analysis (PCA) score plot in positive ion mode. (D) PCA score plot in negative ion mode. (E) Orthogonal partial least squares discriminant analysis (OPLS-DA) score plot in positive ion mode. (F) OPLS-DA permutation test plot in positive ion mode. (G) OPLS-DA score plot in negative ion mode. (H) OPLS-DA permutation test plot in negative ion mode. Group 1 (pre-defined core high-quality production regions): PTW, QG, NFS, DJS, MYX, and FGS; Group 2 (pre-defined general production regions): NC, SKC, YLZ, and HKC; QC: quality control sample pool employed throughout the metabolomics analytical workflow.

In positive ion mode, 17,184 ion features were detected, whereas 14,710 were identified in negative ion mode. Base peak chromatograms revealed that all samples exhibited strong signal intensities, well-resolved chromatographic peaks, and high peak capacities, indicating effective chromatographic separation and high-quality data, which supported reliable metabolite identification and quantification (Fig. 4A, B). PCA revealed notable distinctions in nonvolatile metabolite profiles among samples from different production regions. In positive ion mode (Fig. 4C), PC1 and PC2 accounted for 16.29% and 12.33% of the observed variance, respectively. Conversely, in negative ion mode (Fig. 4D), they explained 17.15% and 12.91% of the variance, respectively. This indicated unique metabolic diversity and suggests potential geographical clustering. To further characterize the metabolic divergence and identify key DAMs, OPLS-DA models were applied. Results from permutation tests confirmed model reliability: in positive ion mode, R^2^X = 0.286, R^2^Y = 0.988, Q^2^ = 0.893 (Fig. 4E, F); in negative ion mode, R^2^X = 0.293, R^2^Y = 0.991, Q^2^ = 0.896 (Fig. 4G, H). These parameters indicated robust explanatory power, excellent predictive accuracy, and high stability, thereby validating the significant metabolic differences among origins.

**Fig. 5:**
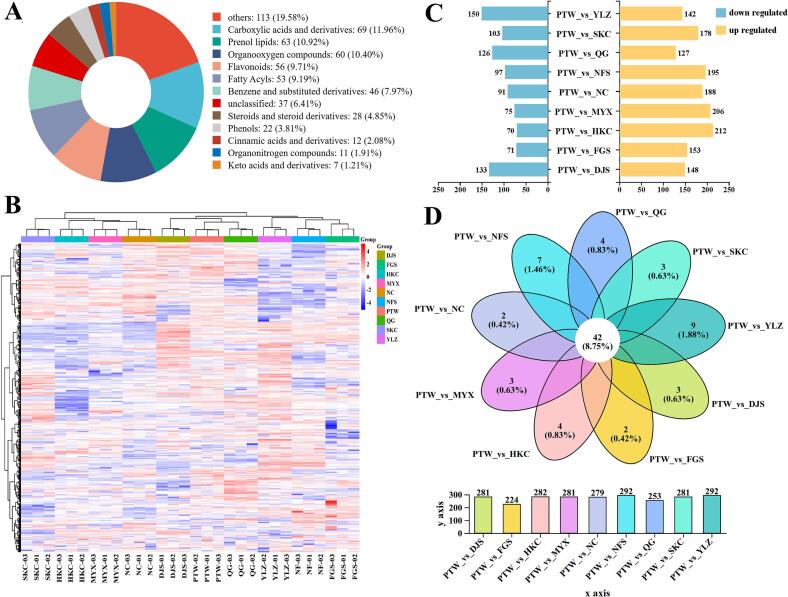
Identification and analysis of *Lithocarpus litseifolius* differential metabolites, following LC-MS/MS detection, across 10 sampling locations. (A) Pie chart illustrating the classification of metabolites. (B) Heatmap analysis of metabolites identified in samples across 10 sampling locations. (C) Statistical analysis of differential metabolites across different comparison groups. (D) Quantitative analysis of specifically differential metabolites between the *L. litseifolius* sample groups from different sampling locations.

A total of 577 metabolites were identified in all samples. The main categories included carboxylic acids and derivatives (*n* = 69, 11.96%), prenol lipids (*n* = 63, 10.92%), organooxygen compounds (*n* = 60, 10.40%), flavonoids (*n* = 56, 9.71%), fatty acyls (*n* = 53, 9.19%), and benzene and substituted derivatives (*n* = 46, 7.97%; Table S1, Fig. 5A). Among flavonoids, the most common types were flavonoid glycosides, flavans, flavones and isoflavonoid O-glycosides. Additionally, several bioactive DHCs—such as phlorizin, trilobatin, and phloretin—were detected, suggesting their potential physiological properties, including antioxidant, anti-inflammatory, and hypoglycemic activities. Unsupervised hierarchical clustering of metabolite profiles grouped the 10 origins into two main clusters: Cluster I (SKC, HKC, NC, and MYX) and Cluster II (DJS, PTW, QG, YLZ, NF, and FGS) (Fig. 5B). This clustering pattern aligns closely with the established delineation of premium production regions and demonstrates a statistically significant positive spatial autocorrelation with geographical proximity. This suggests that abiotic environmental drivers—including climatic variables and edaphic properties—play a critical role in the regional divergence observed in metabolic profiles. The Mantel test identified a significant positive correlation between pairwise metabolic dissimilarities and geographical distances across samples (*r* = 0.412, *P* = 0.0001; Fig. S2A).

To quantify the independent explanatory power of geographic distance on metabolic variation, dbRDA was performed with Euclidean distance derived from GPS coordinates as the sole constrained predictor (Fig. S2B). Geographic distance accounted for 21.7% of the total variation in the Hellinger-transformed metabolite profile (adjusted *R*^*2*^ = 0.217, *P* < 0.01), indicating that spatial segregation due to sampling location is a key determinant of metabolic divergence in *L. litseifolius*. Furthermore, a comprehensive dbRDA model incorporating edaphic properties (e.g., soil available nutrients, pH) and climatic variables (e.g., MAT, MAAP, MAWS) significantly influenced metabolite profiles (*P* < 0.01), confirming that site-specific environmental conditions exert a strong and statistically robust impact on metabolic composition (Fig. S2C).

#### 3.3.2 Differentially accumulated metabolites

DAMs were screened using thresholds of *P* ≤ 0.05 and VIP ≥ 1.0, with PTW as the control group. Pairwise comparisons revealed substantial changes in more and less abundant metabolites (Fig. 5C). Venn diagram analysis identified 480 DAMs, including 42 commonly altered metabolites, predominantly amino acids, peptides, analogs; flavonoid glycosides; and triterpenoids (Fig. 5D). The comparison between PTW and YLZ yielded the highest number of DAMs (*n* = 292) and the most unique DAMs (*n* = 9), whereas the PTW and FGS comparison produced the fewest (*n* = 224) DAMs, including only two unique DAMs.

**Fig. 6:**
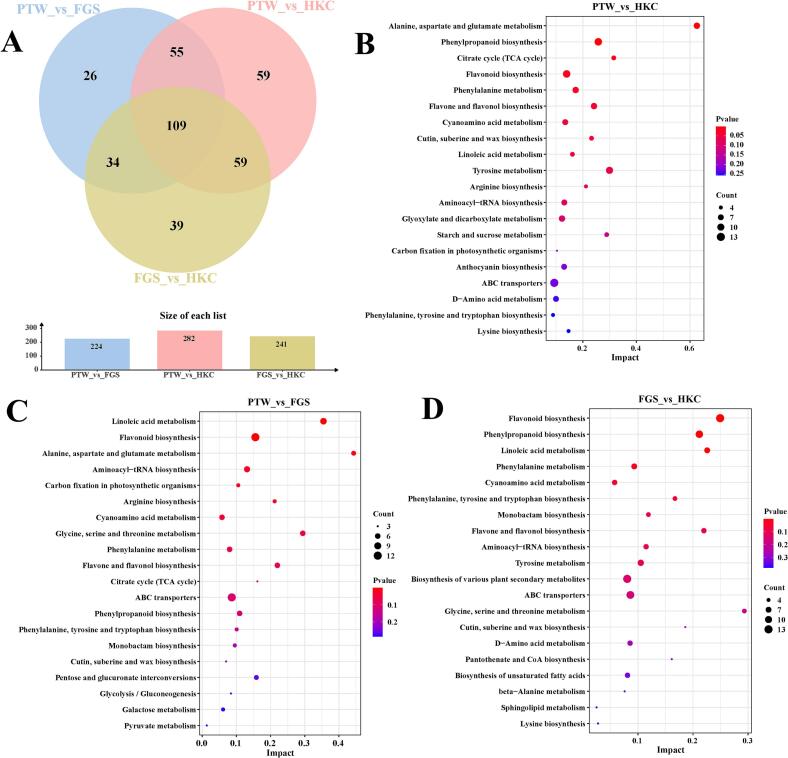
Venn diagram (A) and Kyoto Encyclopedia of Genes and Genomes enrichment analysis of the differential metabolites for the (B) PTW_vs_HKC, (C) PTW_vs_FGS, and (D) FGS_vs_HKC comparisons.

Based on integrated metabolite quantification and a biochemical quality assessment, PTW samples exhibited the highest overall quality owing to their superior levels of bioactive constituents, followed by FGS (moderate quality), whereas HKC samples displayed the lowest quality. Therefore, PTW was selected as the reference group for pairwise comparisons with HKC and FGS to systematically elucidate the differences in secondary metabolism. In total, 109 DAMs were shared among the three groups, primarily categorized as carbohydrates and carbohydrate conjugates; amino acids, peptides, and analogues; linoleic acids and derivatives; and flavonoid glycosides. Specific DAMs included 26 unique to PTW versus FGS, 59 unique to PTW versus HKC, and 39 unique to FGS versus HKC (Fig. 6A).

In the PTW versus HKC comparison, 282 DAMs were identified (212 increased and 70 decreased in PTW). The more abundant compounds in PTW were enriched in flavonoids (e.g., phlorizin, kaempferide, luteolin 7-glucoside, hesperetin, astragalin, pinocembrin, kaempferol, isorhamnetin, procyanidin B2, and isoquercitrin) and phenolic acids (e.g., m-cresol, homovanillic acid, 3,4-dihydroxyphenylglycol, dopamine, 2-methoxy-4-vinylphenol, isoeugenol, and p-hydroxymandelic acid). In contrast, coumarins and their derivatives, including esculetin, aflatoxin G2, and esculin were predominantly decreased. KEGG pathway enrichment analysis revealed 20 significantly enriched pathways (Fig. 6B): alanine, aspartate, and glutamate metabolism (ath00250); phenylpropanoid biosynthesis (ath00940); citric acid cycle (TCA cycle, ath00020); flavonoid biosynthesis (ath00941); phenylalanine metabolism (ath00360); and flavone and flavonol biosynthesis (ath00944).

In the PTW versus FGS comparison, 224 DAMs were detected (153 were increased and 71 were decreased in PTW). Seventeen flavonoids, 16 fatty acyls, and 16 prenol lipids were significantly decreased in the FGS group, whereas 10 organooxygen compounds and 9 carboxylic acids and derivatives were markedly increased. Enrichment analysis revealed 20 top-ranked pathways (Fig. 6C), including linoleic acid metabolism (ath00591); flavonoid biosynthesis (ath00941); alanine, aspartate, and glutamate metabolism (ath00250); aminoacyl-tRNA biosynthesis (ath00970); and carbon fixation in photosynthetic organisms (ath00710).

In the FGS versus HKC comparison, 241 DAMs were identified (140 increased and 101 decreased). The decreased metabolites in the FGS group included 13 amino acids, peptides, and analogs; 5 carbohydrates and carbohydrate conjugates; 5 hydroxycinnamic acids and derivatives; and 4 alcohols and polyols. Conversely, 18 organooxygen compounds, 15 prenol lipids, 14 flavonoids, and 12 phenols were significantly increased. The KEGG analysis indicated that the top 20 enriched pathways were primarily associated with plant flavonoid biosynthesis and phenylpropanoid metabolism (Fig. 6D).

To identify robust metabolite markers distinguishing core high-quality production regions from general production areas, differential metabolite profiling was performed between two pre-established sample groups—core high-quality regions (Group 1) and general regions (Group 2). A total of 44 significantly DAMs were detected, with 30 upregulated and 14 downregulated in Group 1 relative to Group 2 (Fig. S3A). Among the top 20 DAMs ranked by VIP score (VIP > 2.0; Fig. S3B), the five highest-ranking metabolites were bilobalide A, phytosphingosine, uridine, xanthine, and 13-L-hydroperoxylinoleic acid (VIP > 2.2). These metabolites were predominantly classified into six chemical classes: benzene and substituted derivatives, prenol lipids, fatty acyls, organooxygen compounds, phenols, and flavonoids. KEGG pathway enrichment analysis demonstrated significant overrepresentation in “metabolic pathways” (ko01100) and “biosynthesis of secondary metabolites” (ko01110) (FDR < 0.05; Fig. S3C).

### 3.4 Non-targeted metabolomic analysis based on GC–MS

**Fig. 7:**
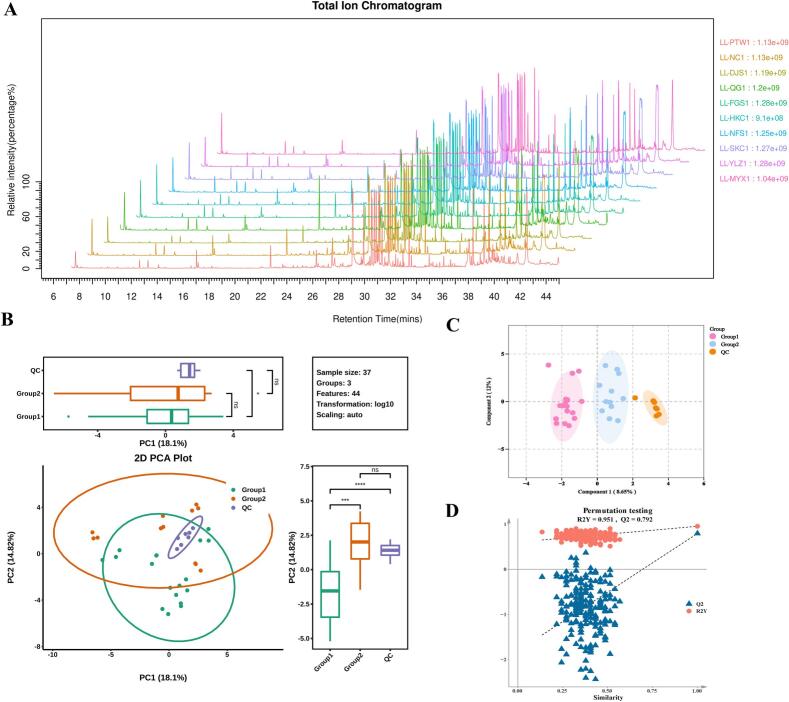
Multivariate statistical analysis of *Lithocarpus litseifolius* metabolites, following GC–MS detection, across 10 sampling locations. (A) Typical total ion chromatogram of representative samples. (B) Principal component analysis (PCA) score plot. (C) Orthogonal partial least squares discriminant analysis (OPLS-DA) score plot. (D) OPLS-DA permutation test plot. Group 1 (pre-defined core high-quality production regions): PTW, QG, NFS, DJS, MYX, and FGS; Group 2 (pre-defined general production regions): NC, SKC, YLZ, and HKC; QC: quality control sample pool employed throughout the metabolomics analytical workflow.

GC–MS combined with derivatization was used to analyze non-volatile metabolites in young leaves of *L. litseifolius* collected from 10 distinct regions in Jiangxi Province (Fig. 7A). In total, 44 metabolites were identified, including 10 organooxygen compounds, 9 carboxylic acids and derivatives, 1 fatty acyls, 1 hydroxy acids and derivatives, 1 pyrimidine nucleosides, and 22 other compounds (Table S2). PCA was performed to assess differences in the overall metabolic profile across geographical regions (Fig. 7B). PCA score plots revealed tight clustering of biological replicates from the same region, indicating high reproducibility and analytical method stability. In contrast, samples from different regions exhibited clear separation, suggesting significant geographical variation in metabolite compositions. Notably, samples from the core high-quality production regions (Group 1) and general production regions (Group 2) exhibit partial spatial overlap in clustering, consistent with their adjacent geographical locations, further supporting the influence of local environmental factors on metabolite accumulation in *L. litseifolius*.

**Fig. 8:**
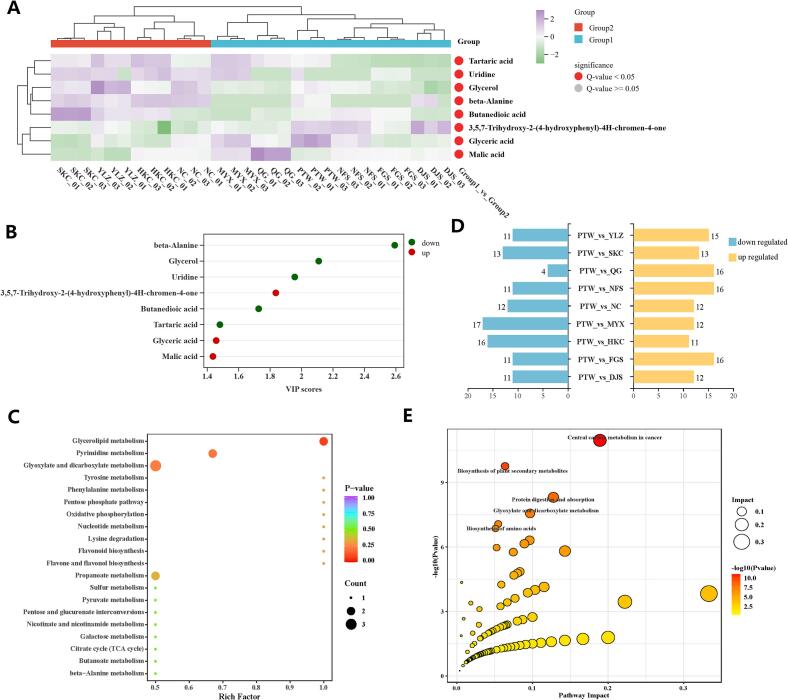
Analysis of *Lithocarpus litseifolius* metabolites, following GC–MS detection, across 10 sampling locations. (A) Heatmap analysis of differentially accumulated metabolites (DAMs) of *L. litseifolius* from 10 geographic locations. (B) Variable importance in projection (VIP) score plot for DAMs. (C) Kyoto Encyclopedia of Genes and Genomes (KEGG) pathway enrichment analysis of DAMs with network visualization. (D) Statistical analyses of the number of differential metabolites between *L. litseifolius* sampling groups from different sampling locations. (E) KEGG enrichment analysis of differential metabolites for the PTW_vs_HKC comparison.

To further characterize region-specific metabolic differences, OPLS-DA was applied to construct a discriminant model (Fig. 7C). The model achieved R^2^X = 0.512, R^2^Y = 0.951, and Q^2^ = 0.792, demonstrating its strong explanatory power and reliable predictive performance (Fig. 7D). This confirmed its suitability for identifying DAMs. Based on *P* ≤ 0.05 and VIP ≥ 1, eight statistically significant DAMs were identified between core high-quality production regions (Group 1) and general production regions (Group 2). Hierarchical clustering of these DAMs separated the 10 regional samples into two Cluster I (SKC, HKC, YLZ, and NC) and Cluster II (FGS, QG, MYX, NFS, PTW, and DJS; Fig. 8A). This classification pattern aligns with the clustering trend observed in the LC-MS/MS analysis and the pre-defined classification of core high-quality and general production areas, confirming the discriminatory power and biological robustness of the selected DAMs as markers for tracing origin.

Given that beta-alanine and glycerol ranked highest in VIP score (> 2.0), they were considered top-tier discriminative metabolites for geographical origin (Fig. 8B). KEGG pathway enrichment analysis revealed that these DAMs were predominantly enriched in key metabolic pathways, including “Metabolic pathways” (ko01100), “Biosynthesis of secondary metabolites” (ko01110), “Glyoxylate and dicarboxylate metabolism” (ko00630), “Pyrimidine metabolism” (ko00240), and “Propanoate metabolism” (ko00640, Fig. 8C). This suggests that geographical origin may influence the chemical quality of *L. litseifolius* by regulating central carbon metabolism and specialized (secondary) metabolic networks.

Using PTW as the control group, each of the nine pairwise comparisons identified 23–29 DAMs (Fig. 8D), highlighting the substantial heterogeneity in metabolic regulation across regions. In the PTW versus HKC comparison, 27 DAMs were detected, comprising 16 more abundant and 11 less abundant metabolites primarily classified as amino acids, peptides and analogs, and carbohydrates and carbohydrate conjugates. KEGG enrichment analysis indicated significant enrichment of certain pathways: plant secondary metabolite biosynthesis; protein digestion and absorption; glyoxylate and dicarboxylate metabolism; and amino acid biosynthesis (Fig. 8E).

### 3.5 Rhizosphere soil characteristics in different origins

To objectively evaluate the influence of the geographical origin on metabolite accumulation in *L. litseifolius*, we systematically analyzed and compared the fundamental physicochemical properties of rhizosphere soils across different production regions. Fig. S4 presents the pH, OM, HN, AP, AK, AZn, ACu, AFe, EMn, and EMg contents in soils from the 10 sampling sites. Rhizosphere soil OM levels were relatively high in the FGS, SKC, and HKC sites. HN content was lower in the YLZ-, NF-, and PTW-origin samples, whereas it was markedly elevated in the QG, MYX, and FGS samples. AP concentrations in the FGS-, SKC-, and PTW-region samples were significantly higher than those in the other regions, suggesting enhanced phosphorus availability. In contrast, AK levels in the YLZ, HKC, and NC samples were significantly reduced, exhibiting notable inter-origin variations, which may constrain plant potassium uptake and utilization. With respect to micronutrients, AZn was significantly enriched in the SKC and HKC samples but substantially depleted in the DJS and FGS samples. ACu was elevated in PTW, HKC, and NF samples but was comparatively lower in the SKC and FGS samples. AFe was significantly higher in SKC, FGS, and MYX samples but was markedly deficient in DJS and NF samples. EMn was significantly increased in QG and PTW samples yet remained low in DJS and FGS samples. EMg was significantly greater in the SKC, MYX, and NC samples, whereas it was significantly diminished in the PTW and YLZ samples. Collectively, these findings demonstrate pronounced spatial heterogeneity in rhizosphere soil nutrient profiles across different geographical origins, which may critically influence *L. litseifolius* growth, as well as the biosynthesis and secondary metabolite accumulation.

### 3.6 Climatic factors in different production regions

To evaluate the influence of climatic factors on variations in *L. litseifolius* quality, meteorological data were collected from 10 production regions over the 2022–2024 period (Fig. S5). The PTW region exhibited the highest annual mean temperature, whereas the HKC region had the lowest, indicating pronounced interregional thermal differences. With respect to precipitation, FGS, DJS, and NC regions had relatively high annual rainfall, whereas HKC and SKC regions had significantly lower levels. Regarding solar radiation and wind speed, SKC, QG, and PTW had longer mean annual sunshine durations, suggesting greater availability of photosynthetically active radiation, whereas HKC, FGS, and YLZ experienced shorter mean annual sunshine durations. In terms of wind, the NF, PTW, and DJS regions had higher annual mean wind speeds, with HKC showing the lowest value, which is indicative of reduced atmospheric circulation. Atmospheric pressure data indicated an elevated MAAP in the QG, PTW, and NC regions, whereas the YLZ and FGS regions had lower values. Analyses of humidity and soil temperature revealed that the YLZ, QG, and HKC regions had the highest MAH, whereas PTW had the lowest, reflecting substantial regional variation in atmospheric moisture conditions. At a soil depth of 20 cm, the NF, FGS, and HKC regions had the highest mean annual ground temperatures, whereas YLZ had the lowest, implying a comparatively weaker root-zone thermal environment. Collectively, these findings highlight the significant spatial heterogeneity in climatic conditions across production regions, which may critically influence growth, development, and secondary metabolite accumulation in *L. litseifolius*.

### 3.7 Multi-platform metabolite integration analysis and identification of origin-associated quality markers

**Fig. 9:**
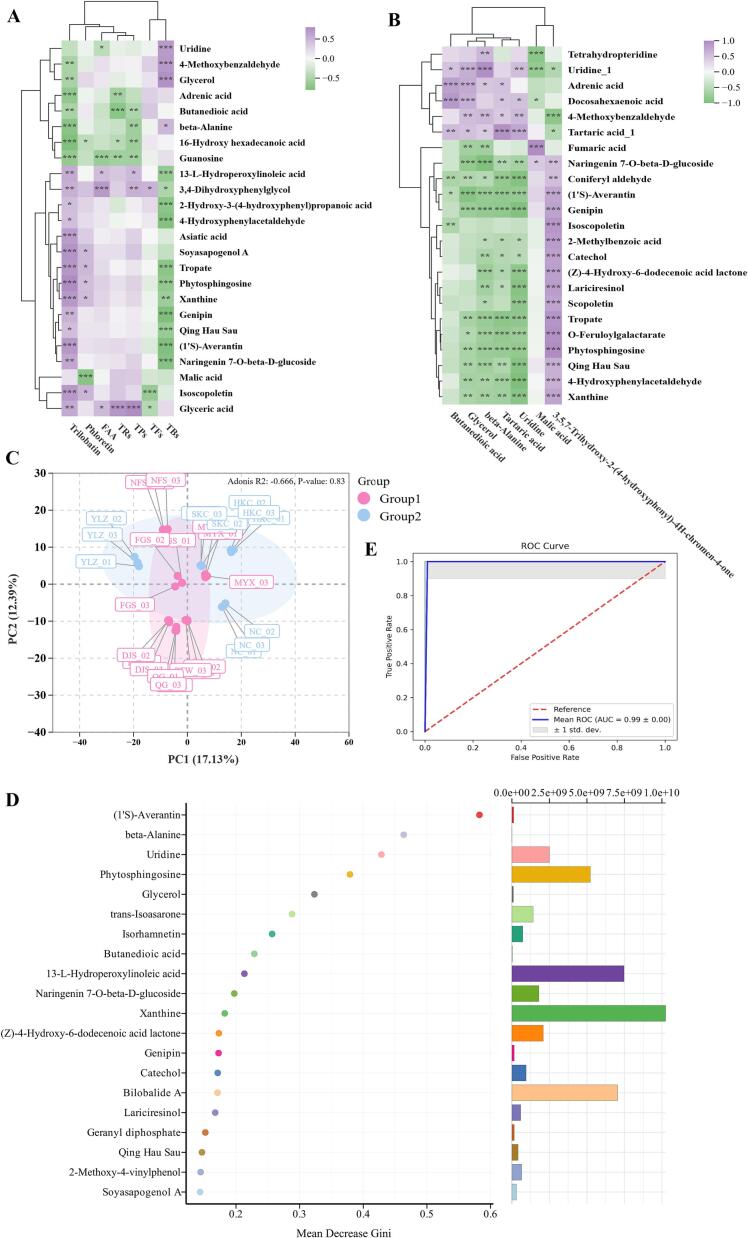
Cross-platform metabolite correlation analysis and random forest modeling results. (A) Spearman correlation analysis between quality-related indicators and differentially accumulated metabolites (DAMs) identified by non-targeted metabolomics; (B) Inter-platform Spearman correlation of DAMs identified by LC-MS/MS and GC–MS; (C) Principal Component Analysis (PCA) score plot derived from integrated LC-MS/MS and GC–MS data; (D) Feature importance ranking from the random forest model; (E) Receiver Operating Characteristic (ROC) curve generated by the random forest classifier. TPs, tea polyphenol; TFs, theaflavins; TBs, theabrownins; TRs, thearubigins; FAA, free amino acid. Group 1 (pre-defined core high-quality production regions): PTW, QG, NFS, DJS, MYX, and FGS; Group 2 (pre-defined general production regions): NC, SKC, YLZ, and HKC; QC: quality control sample pool employed throughout the metabolomics analytical workflow.

To systematically elucidate the metabolic regulatory basis underlying quality variation in *L. litseifolius* across geographical origins in Jiangxi Province, non-targeted metabolomic profiles, targeted quantitative data for DHCs (trilobatin, phloretin, phlorizin), and conventional biochemical parameters (TF, TPs, TFs, TRs, and TBs, FAA) were integrated to construct a multi-dimensional Spearman correlation network. This approach facilitated the elucidation of biologically meaningful associations between metabolites and quality traits, thereby strengthening the evidence supporting discrimination of geographical origins. Notably, significant cross-platform correlations (|*r*| ≥ 0.55, *P* < 0.01, FDR-corrected, Fig. 9A) were observed among DAMs identified via non-targeted metabolomics and key quality-related compounds across Group 1 (core high-quality production areas: PTW, QG, NFS, DJS, MYX, and FGS) and Group 2 (general production areas: NC, SKC, YLZ, and HKC). Specifically, trilobatin exhibited strong positive correlations with (1′*S*)-averantin, asiatic acid, isoscopoletin, phytosphingosine, soyasapogenol A, tropate, and xanthine (|*r*| > 0.60), and strong negative correlations with guanosine, adrenic acid, beta-alanine, and 16-hydroxy hexadecanoic acid (|*r*| > 0.60). TBs demonstrated significant positive associations with 4-methoxybenzaldehyde, glycerol, and uridine, but negative associations with qing hau sau, 13-L-hydroperoxylinoleic acid, phytosphingosine, tropate, 2-hydroxy-3-(4-hydroxyphenyl) propanoic acid, naringenin 7-O-beta-D-glucoside, 4-hydroxyphenylacetaldehyde, (1′*S*)-averantin, and genipin (|*r*| > 0.60). Both TPs and TRs exhibited strong positive correlations with glyceric acid; TRs were also significantly negatively correlated with butanedioic acid, while phloretin was significantly negatively correlated with fumaric acid and malic acid (|*r*| > 0.55).

Cross-platform correlation analysis between metabolites detected by GC–MS and LC-MS/MS revealed that beta-alanine, tartaric acid, and uridine (GC–MS) were significantly inversely associated with (1′*S*)-averantin, genipin, phytosphingosine, and tropate (LC-MS/MS) (|*r*| > 0.60, *P* < 0.01); tartaric acid and uridine also demonstrated strong inverse relationships with coniferyl aldehyde and O-feruloylgalactarate (|*r*| > 0.60, *P* < 0.01; Fig. 9B). Beta-alanine and glycerol jointly showed robust negative correlations with naringenin 7-O-beta-D-glucoside, while both exhibited strong positive correlations with uridine (|*r*| > 0.60, *P* < 0.01). Moreover, the flavonoid 3,5,7-trihydroxy-2-(4-hydroxyphenyl)-4H-chromen-4-one displayed strong positive correlations with (1′*S*)-averantin, (Z)-4-hydroxy-6-dodecenoic acid lactone, 2-methylbenzoic acid, catechol, penipin, isoscopoletin, lariciresinol, O-feruloylgalactarate, phytosphingosine, scopoletin, tropate, and xanthine, and a robust negative correlation with 4-methoxybenzaldehyde (|*r*| > 0.60, P < 0.01).

To maximize the discriminative power of complementary analytical platforms, low-level data fusion was performed by horizontally concatenating the GC–MS (*n* = 44 metabolites) and the LC-MS (*n* = 577 metabolites) datasets into a unified metabolite matrix. Using predefined geographical groupings, unsupervised PCA and supervised RF classification models were trained in RStudio to assess inter-group metabolic divergence and prioritize origin-discriminatory features. Applying PCA to the integrated dataset significantly enhanced separation between groups relative to single-platform analyses (Fig. 9C). RF feature importance analysis identified the top 20 metabolites most effective in distinguishing production regions (Fig. 9D).

By integrating variable importance (mean decrease in Gini impurity) with biological abundance, (Z)-4-hydroxy-6-dodecenoic acid lactone, 13-L-hydroperoxylinoleic acid, bilobalide A, naringenin 7-O-beta-D-glucoside, phytosphingosine, trans-isoasarone, uridine, and xanthine were prioritized as candidate region-specific metabolic markers. The RF classifier trained on multi-platform metabolomic data achieved excellent performance, with an area under the receiver operating characteristic curve (AUC) of 0.99 (Fig. 9E), thus demonstrating that cross-platform data fusion substantially enhances the accuracy and reliability of origin authentication for *L. litseifolius*.

### 3.8 Environmental–metabolite association analysis

A multi-layered framework was created for environmental–metabolite–quality association in *L. litseifolius* by integrating non-targeted metabolomic profiles, targeted core DHC quantification, and key biochemical traits with rhizosphere soil properties (10 parameters) and long-term meteorological data (7 variables).

Mantel tests were conducted to assess global matrix-level correlations among the (i) quality trait matrix (DHCs, FAA, TF, TP, TPs), (ii) DAMs abundance matrix, and (iii) environmental matrix. Results revealed statistically significant positive associations between the quality and metabolic phenotypes and multiple environmental factors (*P* < 0.05; Fig. S6A). Specifically, core quality markers (DHC, FAA, TF, TP, TPs) exhibited significant positive correlations with soil ACu, AK, AP, EMn, AFe, MAT, MAAP, and MAH) (|*r*| > 0.11, *P* < 0.05). At the metabolome level, overall DAMs abundance correlated significantly and positively with AK, AZn, and EMn (|*r*| > 0.24, *P* < 0.01; Fig. S6B), while GC–MS-detected DAMs exhibited marked positive relationships with EMg, MASD, MAAP, and MAGT (|*r*| > 0.18, *P* < 0.05).

To identify metabolite-specific environmental linkages, Spearman rank correlation analyses were conducted between individual DAMs and each environmental variable (Fig. S6C). The primary findings were as follows: (i) 4-methoxybenzaldehyde and tartaric acid displayed strong positive correlations with AZn (|*r*| > 0.60, *P* < 0.01); (ii) (1′*S*)-averantin, genipin, isorhamnetin, O-feruloylgalactaric acid, qinghau sau, and tropate were strongly and negatively correlated with AZn (|*r*| > 0.75, *P* < 0.01); (iii) (1′*S*)-averantin and tropate positively correlated with AK (|*r*| > 0.60, *P* < 0.01), while 2-deoxyecdysone, beta-alanine, and glycerol negatively correlated with AK (|*r*| > 0.60, *P* < 0.01); (iv) phenylacetic acid and diaminopimelic acid correlated positively with AP (|*r*| > 0.60, *P* < 0.01); (v) docosahexaenoic acid and prostaglandin I₂ negatively correlated with MAAP and MAGT, respectively (|*r*| > 0.60, *P* < 0.01).

Collectively, these findings indicate that the spatial heterogeneity of secondary metabolites in *L. litseifolius* is governed by the synergistic regulation by soil mineral bioavailability—particularly AK, AZn, ACu, EMn, AFe, and EMg—as well as climatic gradients characterized by temperature, precipitation, humidity, wind speed, and ground temperature. This dual regulatory mechanism functions through integrated physiological–biochemical responses, including nutrient transporter expression, redox-sensitive enzyme modulation, and phytohormone-mediated signaling, thereby establishing a robust ecological–metabolic foundation for terroir-driven quality differentiation.

## 4 Discussion

The quality attributes of medicinal plants arise from the integrated effects of genotype, ecological environment, and cultivation management practices (Yu, Liao, et al., 2025). Genotype–environment (G × E) interactions represent the primary drivers of systematic inter-regional variation in phytochemical composition, pharmacological potency, and clinical therapeutic outcomes (Wang et al., 2025). In this study, *L. litseifolius* samples were collected from 10 major production areas across Jiangxi Province. Key quality traits—including DHCs, TF, TPs, TP, and FAA—were quantified, and their spatial distribution patterns systematically analyzed. The results demonstrated significant geographic variation across all assessed traits, facilitating robust stratification into core high-quality and conventional production zones, thereby providing strong empirical support for the origin-based quality classification framework. Notably, large-scale cultivation relies primarily on germplasm from Hengfeng, Jinggangshan, and Dexing. Population genetic analyses using molecular markers confirmed minimal genetic divergence among these sources (Luo, 2025), indicating pronounced germplasm homogeneity. Collectively, these findings underscore that environmental heterogeneity, rather than genetic diversity, is the predominant determinant of quality differentiation in Jiangxi's *L. litseifolius*. Higher concentrations of DHCs, TF, and TP in core high-quality regions indicate stronger potential bioactivities, including hypoglycemic, antioxidant, antihypertensive, and anti-inflammatory effects (Liu et al., 2025; Shang et al., 2022). Therefore, the geographical variation in metabolite profiles observed in this study not only reflects quality divergence but also implies differences in potential health benefits. These findings provide a scientific basis for selecting superior production areas and optimizing cultivation practices.

Mantel tests identified highly significant positive correlations between key quality traits and DAMs, as well as the soil bioavailability of copper, zinc, potassium, phosphorus, manganese, and iron. Copper serves as an obligate cofactor for polyphenol oxidase (PPO) and Cu/Zn-superoxide dismutase (Cu/Zn-SOD), directly mediating phenolic oxidation/polymerization and intracellular redox homeostasis (Akhtar et al., 2025). Elevated ACu concentrations in high-quality zones likely enhance target compound biosynthesis via two biochemical pathways: (i) increased PPO activity redirects phenylpropanoid metabolism toward flavonoid and DHC biosynthetic branches; and (ii) elevated Cu/Zn-SOD activity mitigates oxidative stress, maintaining a favorable intracellular environment for secondary metabolic processes. Additionally, potassium, phosphorus, manganese, and iron exert complementary roles in osmotic regulation, ATP synthesis, mitochondrial electron transport, and metalloenzyme formation, collectively optimizing rhizosphere biogeochemistry and metabolic resource allocation. This multi-nutrient synergism aligns with previous findings indicating that Cu, Zn, P, and Fe coordinate the regulation of phlorizin and trilobatin glycoside accumulation in *L. litseifolius* (Huang et al., 2025; Yu, Xiong, et al., 2025). Moreover, quality traits exhibited significant positive correlations with MAT, MAAP, and MAH, confirming climate as an independent and critical extrinsic modulator of quality formation. These observations are consistent with established trends in tea, in which low temperatures, high humidity, and pronounced diurnal thermal amplitude at high elevations are associated with increased photosynthetic carbon allocation to specialized metabolites and accelerated biosynthetic activity (Tang et al., 2026).

Untargeted metabolomics analysis revealed robust, reproducible metabolic phenotypic differences between production regions of *L. litseifolius*. Both PCA and OPLS-DA achieved complete class separation between core high-quality and general production zones. Combining LC-MS and GC–MS datasets markedly improved group separation, reflecting enhanced metabolic coverage and analytical orthogonality. Unsupervised hierarchical clustering resolved the 10 sampling locations into two highly cohesive, statistically distinct clusters that aligned closely with the predefined quality classification—core high-quality versus general production areas—and exhibited strong spatial autocorrelation. dbRDA revealed that geographic distance alone accounted for 21.7% of total metabolic variance, supporting the environmental metabolomics paradigm: neighboring sites experience convergent abiotic (e.g., temperature, precipitation, soil pH) and biotic (e.g., rhizosphere microbiota composition) drivers that coordinately regulate secondary metabolic biosynthesis at the transcriptional and post-transcriptional level, leading to region-specific chemical profiles (Danczak et al., 2020; Li et al., 2020). Following stringent statistical filtering, 44 LC-MS/MS-derived and 8 GC–MS-derived DAMs were identified as reliable candidates for geographical quality authentication.

To prioritize biologically interpretable markers, RF feature selection was applied, combining mean decrease in Gini impurity and normalized peak intensity to rank metabolites. This yielded a concise set of eight metabolites with maximal regional discriminatory power. The AUC reached 0.99, reflecting excellent classification accuracy. However, given the well-documented sensitivity of RF models to small training sets (Belgiu & Drăguţ, 2016) and the modest cohort size (*n* = 30) of the current study, these findings should be considered as hypothesis-generating rather than definitive; further validation requires a larger, geographically stratified cohort and independent external testing.

Cross-platform integrative network analysis revealed functional associations between key bioactive constituents and endogenous metabolic networks. Correlation analyses indicated that trilobatin exhibited significant positive correlations with asiatic acid, isoscopoletin, and soyasapogenol A, but negative correlations with guanosine and 16-hydroxyhexadecanoic acid. Meanwhile, phloretin exhibited a significant negative correlation with fumaric acid and malic acid. This co-accumulation–antagonism pattern reflects competitive interactions for shared metabolic precursors and energy cofactors, regulated by hierarchical coordination across secondary metabolic pathways. Acetyl-CoA serves as a critical metabolic node: as the common entry point for the mevalonate pathway and de novo fatty acid biosynthesis (Li et al., 2025; Zhang, Hao, et al., 2025), its allocation between terpenoid/flavonoid production and lipid synthesis directly affects the reciprocal accumulation dynamics between asiatic acid/trilobatin and 16-hydroxyhexadecanoic acid. Phosphoenolpyruvate functions as a central metabolic branching point, fueling the shikimate pathway to generate phenylalanine—an essential precursor for trilobatin and isoscopoletin synthesis—while also replenishing oxaloacetate through anaplerotic carboxylation, sustaining tricarboxylic acid cycle flux and influencing levels of fumaric acid and malate. Moreover, trilobatin biosynthesis demands substantial cellular energy and reducing power; thus, its robust accumulation competitively limits ATP and NADPH availability for other high-flux processes, including purine nucleotide synthesis (e.g., guanosine/GTP) and mitochondrial oxidative phosphorylation.

Notably, the transcriptional mechanisms underlying the superior chemical phenotype of high-quality production regions remain uncharacterized. To address this, the next phase of our research will employ comparative transcriptomic profiling between premium and standard cultivation zones to systematically delineate the gene regulatory networks that drive specialized biosynthesis and optimized metabolism characteristic of high-quality medicinal plant materials.

## 5 Conclusion

This study provides robust empirical evidence of significant geographic variation in the quality of *L. litseifolius* in Jiangxi Province. In core high-quality production zones, increased concentrations of key bioactive compounds are attributed to the coordinated influence of soil mineral nutrients (ACu, AK, AZn) and climatic factors (MAT, MAH, MAAP). These elements collectively facilitate targeted activation of the phenylpropanoid pathway and flavonoid biosynthesis. A rigorously validated set of eight DAMs—identified through integrated LC-MS/GC–MS profiling and multivariate statistical modeling—demonstrates considerable potential as chemical markers for the authentication of geographical origin and objective quality assessment. Additionally, the “environment–metabolism–phenotype” triadic regulatory network established in this study constitutes the first mechanistic framework clarifying how abiotic environmental gradients direct metabolic programming and influence quality outcomes in *L. litseifolius*. Collectively, this study establishes a scientific foundation and methodological approach for identifying high-quality production areas, standardizing cultivation practices, and safeguarding the authenticity of medicinal and edible plants in China.

## CRediT authorship contribution statement

**Yuling Wang:** Writing – original draft, Methodology, Investigation. **Bing Cao:** Supervision, Conceptualization. **Jianfeng Cheng:** Software, Resources, Data curation. **Mengxing Wang:** Validation, Investigation. **Zixuan Qiu:** Writing – review & editing, Supervision, Funding acquisition. **Wuping Yan:** Writing – review & editing, Resources, Project administration.

## Funding sources

This work was supported by the 10.13039/501100001809National Natural Science Foundation of China [Grant Number 32160364], the Doctoral Start-up Fund of Jiangxi Agricultural University [Grant Number 9232309477], and the Hainan Province Innovative Scientific Research Projects of Postgraduates [Grant Number Qhyb2024-88].

## Declaration of competing interest

The authors declare that they have no known competing financial interests or personal relationships that could have appeared to influence the work reported in this paper.

## Data Availability

Data will be made available on request.

## Appendix A Supplementary data

Supplementary material 1: Supplementary Figures

Supplementary material 2: Quantitative dataset of metabolites
