# Supplementary material for: Phytochemical-based quality and marker identification of sweet tea (Lithocarpus litseifolius [hance] Chun) from various Jiangxi regions
Source: Food Chem X. 2026 May 30;36:104049. doi: 10.1016/j.fochx.2026.104049 (PMC13233576; doi:10.1016/j.fochx.2026.104049)
Supplement: Supplementary material 1: Supplementary Figures [file mmc1.docx]

**Supporting Information for**

## **Phytochemical-based quality and marker identification of sweet tea (*Lithocarpus litseifolius* [Hance] Chun) from various Jiangxi regions**

Yuling Wang^a, b^, Bing Cao^c^, Jianfeng Cheng^b^, Mengxing Wang^b^, Zixuan Qiu^a,^ *, Wuping Yan^b,^ *

^a^School of Breeding and Multiplication (Sanya Institute of Breeding and Multiplication), Hainan University, Sanya 572025, China

^b^School of Agricultural Sciences, Jiangxi Agricultural University, Nanchang 330045, China

^c^Hainan Academy of Agricultural Sciences, Haikou 570203, China

***Corresponding author.**

**Email:** *zixuanqiu@hainanu.edu.cn; yanwuping@jxau.edu.cn*

**
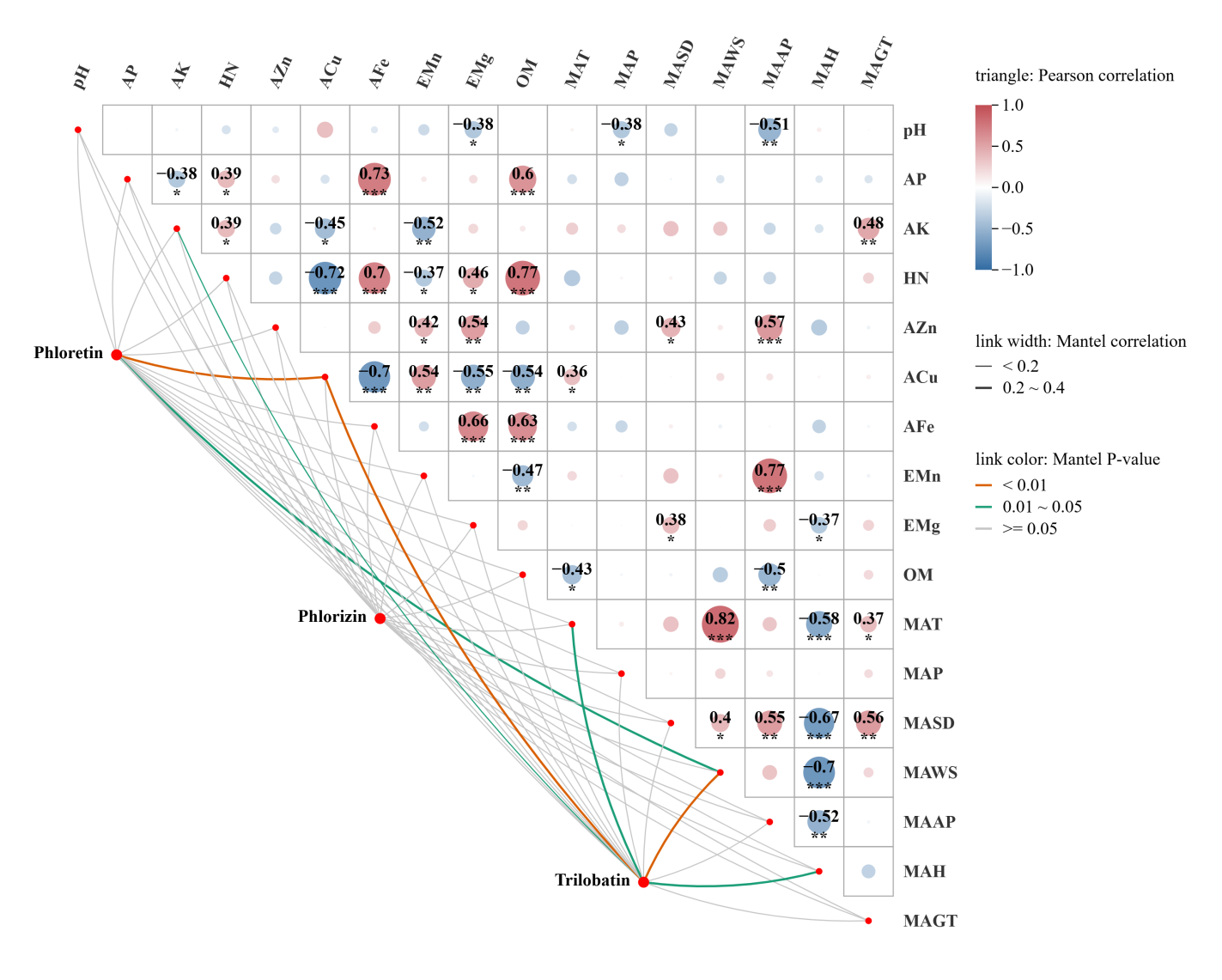
**

**Fig. S1. Mantel test of correlations between the three dihydrochalcone contents and environmental factors.** OM, organic matter; HN, alkali-hydrolyzable nitrogen; AP, available phosphorus; AK, available potassium; AFe, available ferrum; EMn, Exchangeable manganese; ACu, available cuprum; AZn, available zinc; EMg, exchangeable magnesium; MAAP, mean annual air pressure; MAT, mean annual temperature; MAH, mean annual humidity; MAP, mean annual precipitation; MAWS, mean annual wind speed; MASD, mean annual sunshine duration; MAGT, mean annual ground temperature.


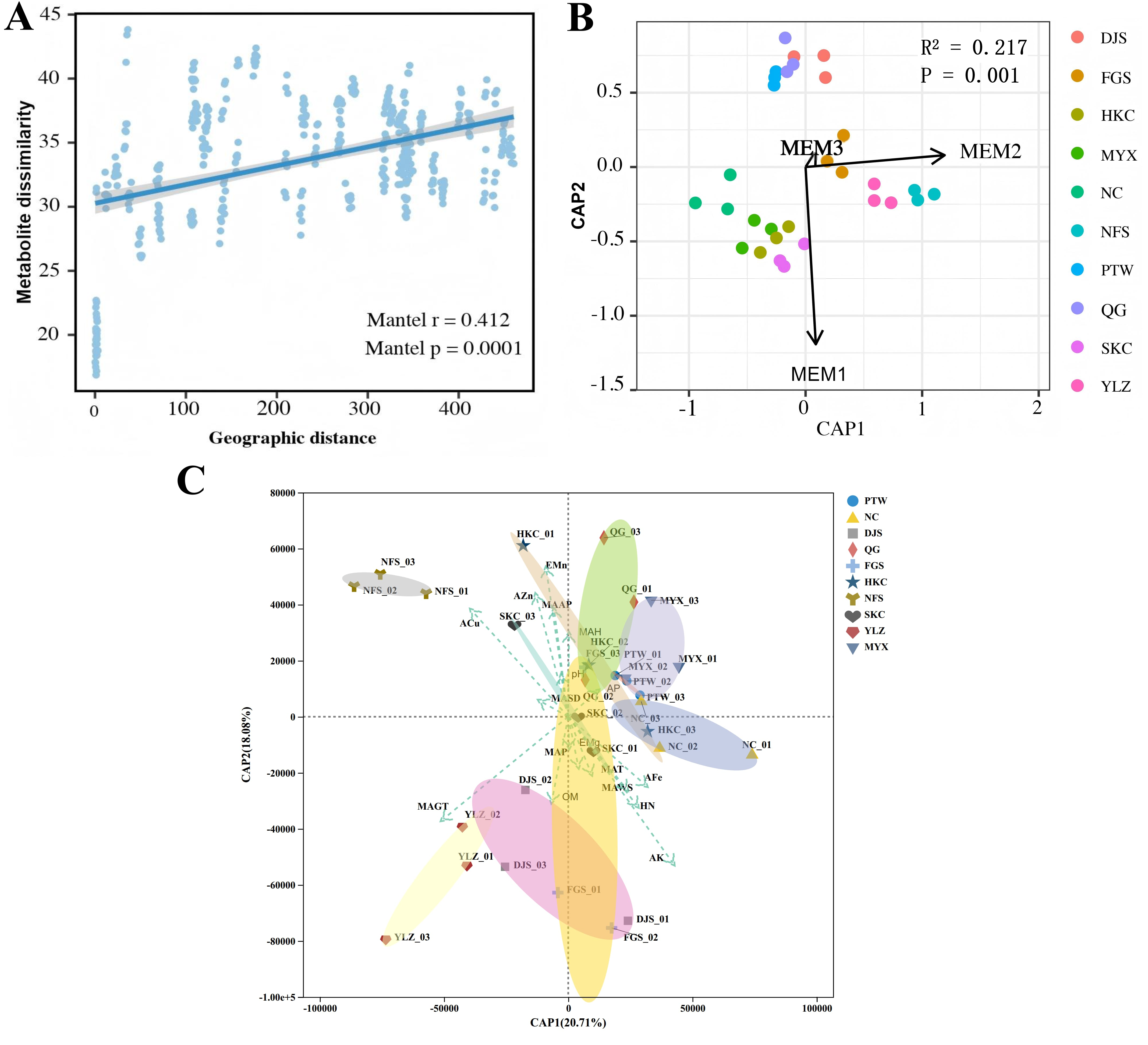


**Fig. S2.** **Mantel test and distance-based redundancy analysis (dbRDA) results.** **(A)** Mantel correlation test between geographical distance and metabolite dissimilarity matrices; **(B)** dbRDA ordination plot constrained by geographical distance; **(C)** dbRDA ordination plot constrained by environmental variables.


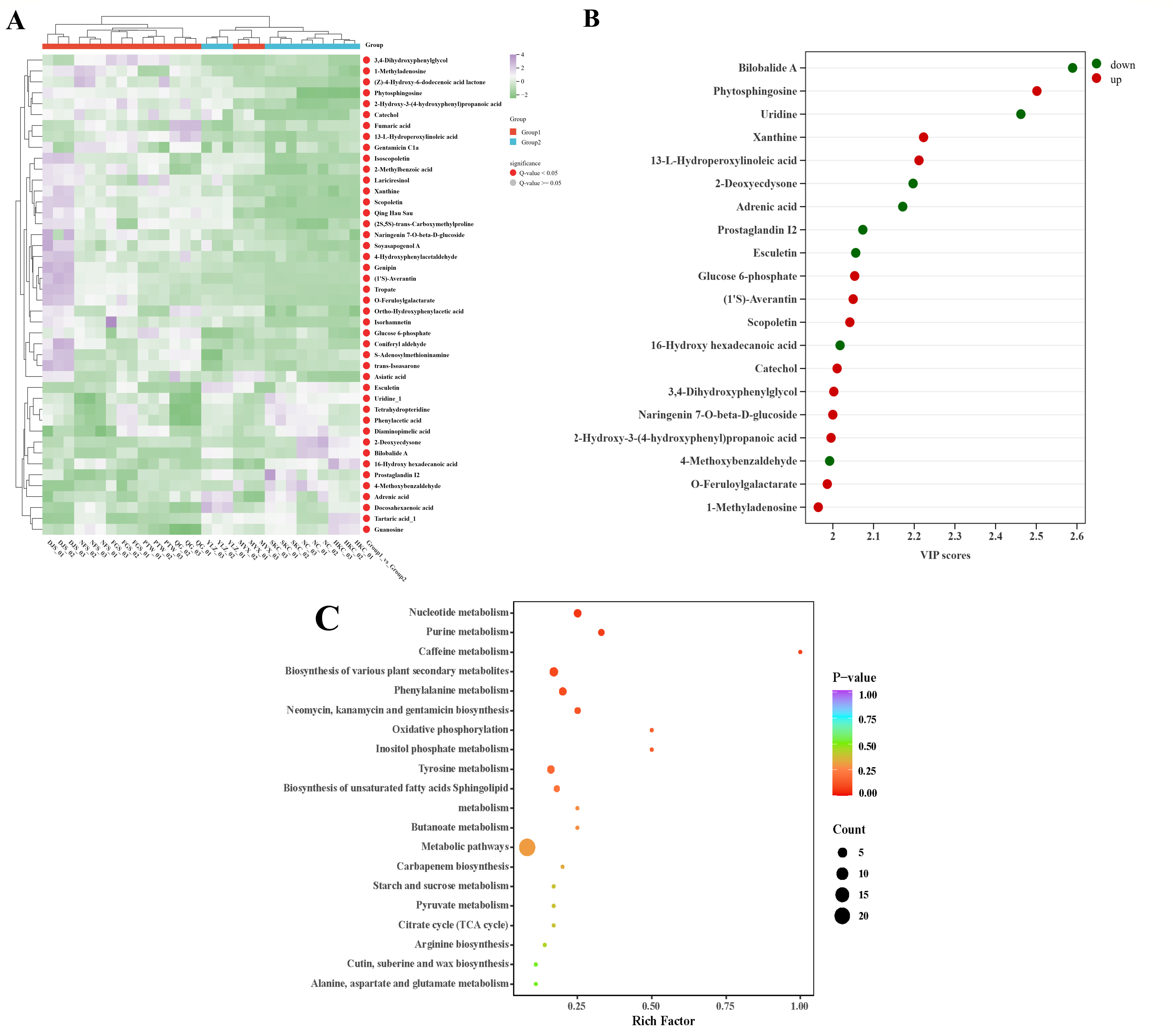


**Fig. S3. Differential accumulation analysis of non-volatile metabolites between high-quality (Group 1) and general production (Group 2) areas.** **(A)** Heatmap analysis of differentially accumulated metabolites (DAMs) of *L. litseifolius* from ten geographic locations. **(B)** Variable importance in projection (VIP) score plot for DAMs. **(C)** Kyoto Encyclopedia of Genes and Genomes (KEGG) pathway enrichment analysis of DAMs with network visualization.


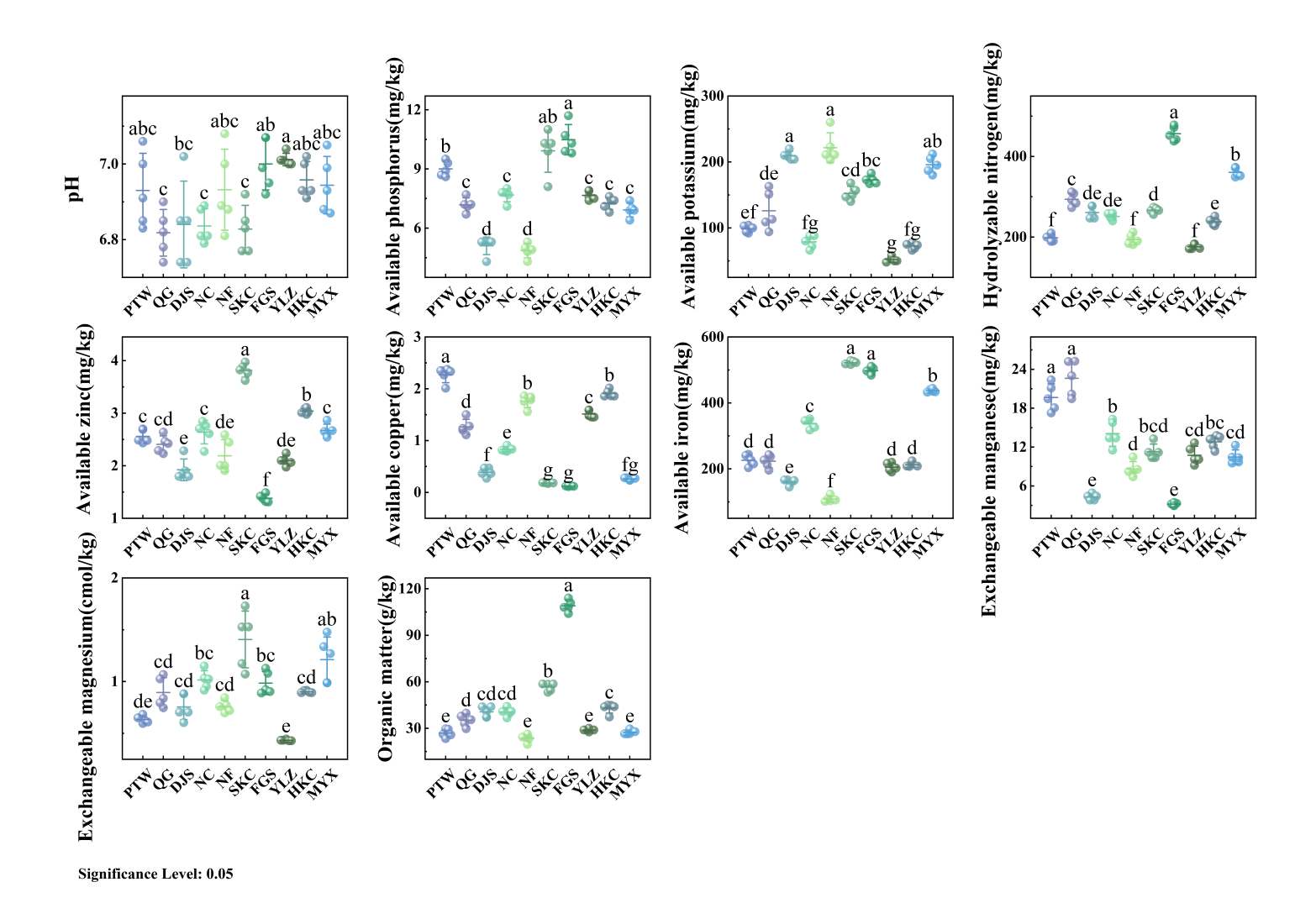


**Fig. S4. Physicochemical properties of the rhizosphere soil at the ten *Lithocarpus litseifolius* sampling locations*.***

**
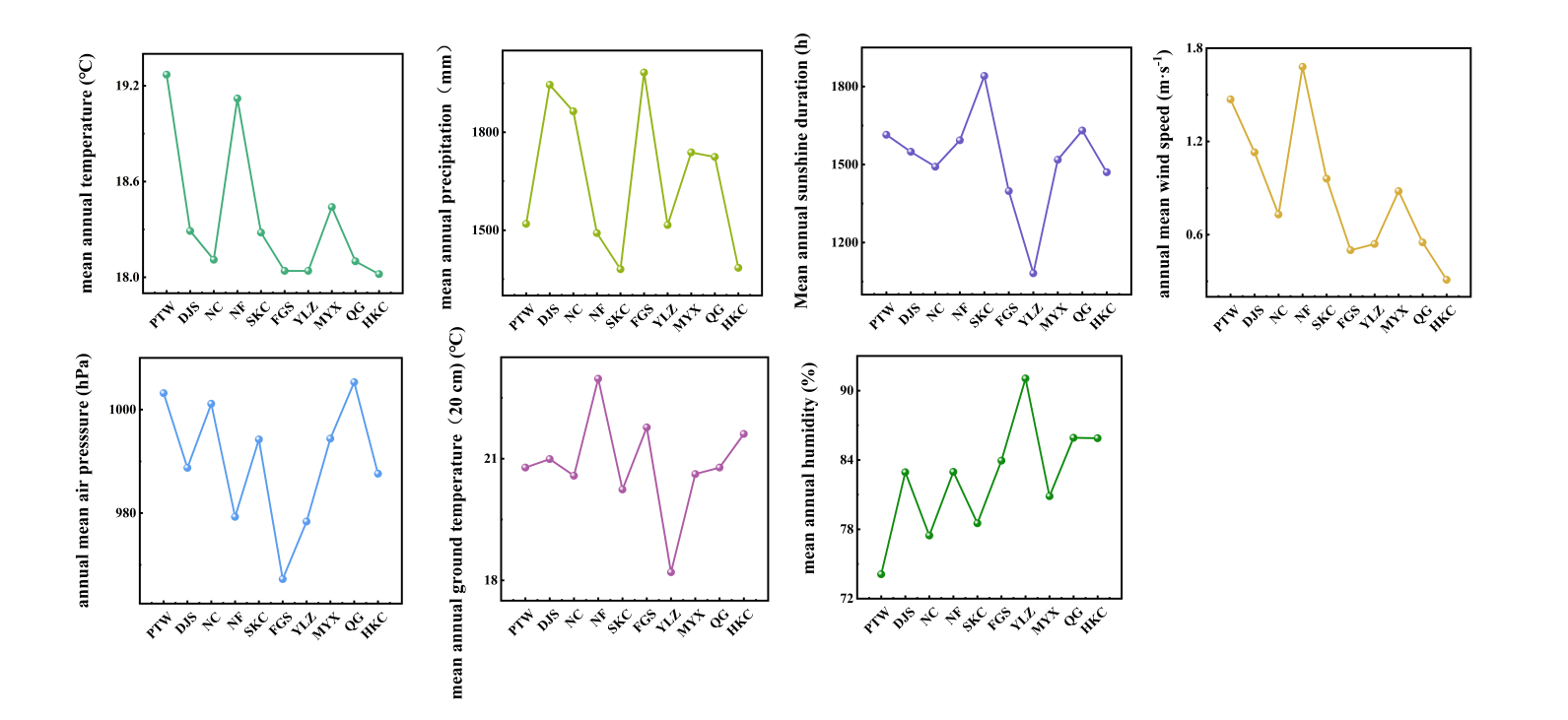
**

**Fig. S5. Meteorological factors at the ten *Lithocarpus litseifolius* sampling locations*.***

***
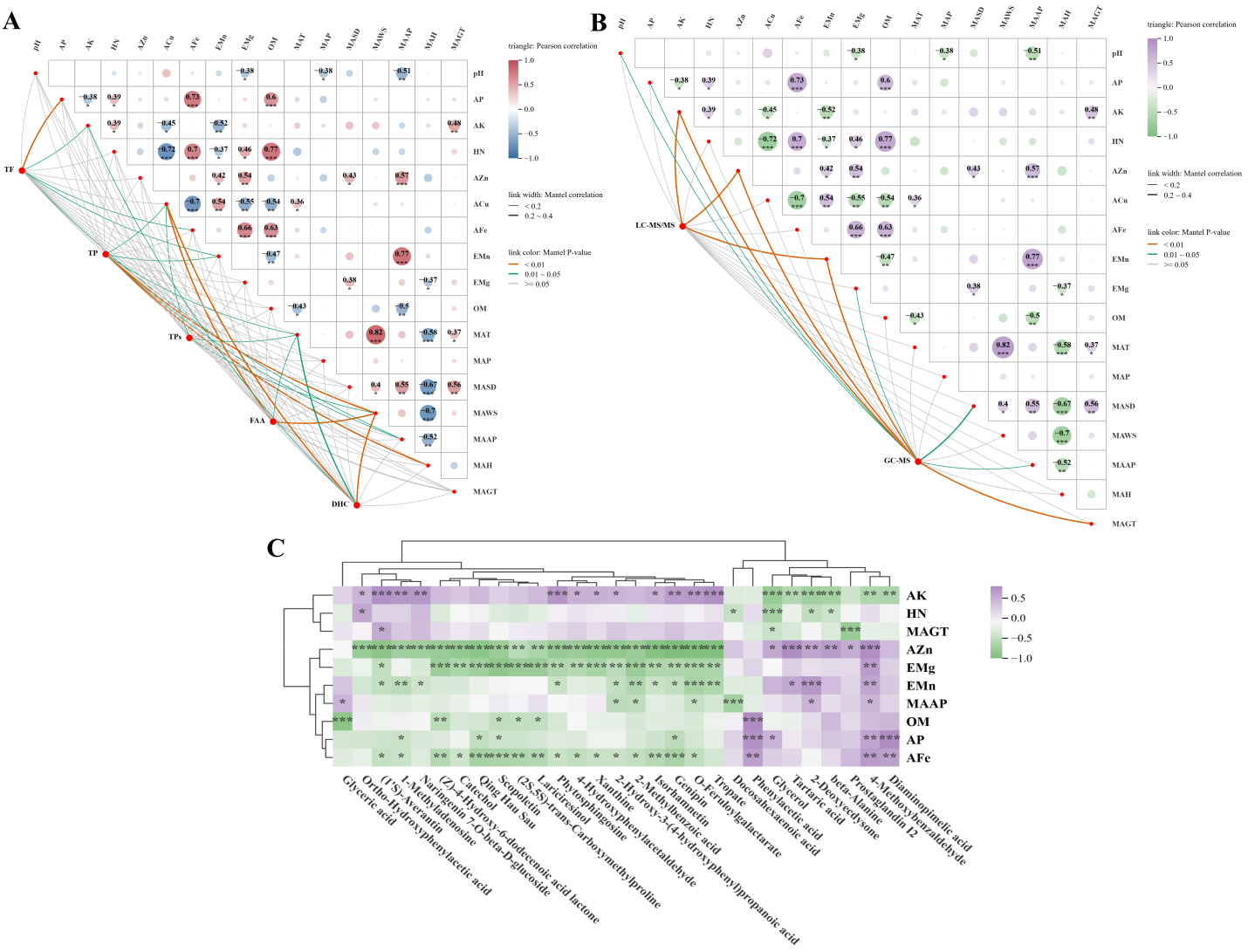
***

**Fig. S6. Correlation analysis among metabolites, quality traits, and environmental factors. (A)** Mantel test between quality traits and environmental factors; **(B)** Mantel test between differentially accumulated metabolites (DAMs) and environmental factors; **(C)** Spearman correlation analysis of DAMs with environmental factors.
